# Combination therapy with pemafibrate (K-877) and pitavastatin improves vascular endothelial dysfunction in dahl/salt-sensitive rats fed a high-salt and high-fat diet

**DOI:** 10.1186/s12933-020-01132-2

**Published:** 2020-09-26

**Authors:** Masatoki Yoshida, Kazufumi Nakamura, Toru Miyoshi, Masashi Yoshida, Megumi Kondo, Kaoru Akazawa, Tomonari Kimura, Hiroaki Ohtsuka, Yuko Ohno, Daiji Miura, Hiroshi Ito

**Affiliations:** 1 Department of Cardiovascular Medicine, Okayama University Graduate School of Medicine, Dentistry and Pharmaceutical Sciences, 2-5-1 Shikata-cho, Kita-ku, Okayama, 700-8558 Japan; 2 Department of Medical Technology, Kawasaki College of Allied Health Professions, Okayama, Japan; 3 Department of Basic and Clinical Medicine, Nagano College of Nursing, Nagano, Japan

**Keywords:** Pemafibrate, Statin, Endothelial function

## Abstract

**Background:**

Statins suppress the progression of atherosclerosis by reducing low-density lipoprotein (LDL) cholesterol levels. Pemafibrate (K-877), a novel selective peroxisome proliferator-activated receptor α modulator, is expected to reduce residual risk factors including high triglycerides (TGs) and low high-density lipoprotein (HDL) cholesterol during statin treatment. However, it is not known if statin therapy with add-on pemafibrate improves the progression of atherosclerosis. The aim of this study was to assess the effect of combination therapy with pitavastatin and pemafibrate on lipid profiles and endothelial dysfunction in hypertension and insulin resistance model rats.

**Methods:**

Seven-week-old male Dahl salt-sensitive (DS) rats were divided into the following five treatment groups (normal diet (ND) plus vehicle, high-salt and high-fat diet (HD) plus vehicle, HD plus pitavastatin (0.3 mg/kg/day), HD plus pemafibrate (K-877) (0.5 mg/kg/day), and HD plus combination of pitavastatin and pemafibrate) and treated for 12 weeks. At 19 weeks, endothelium-dependent relaxation of the thoracic aorta in response to acetylcholine was evaluated.

**Results:**

After feeding for 12 weeks, systolic blood pressure and plasma levels of total cholesterol were significantly higher in the HD-vehicle group compared with the ND-vehicle group. Combination therapy with pitavastatin and pemafibrate significantly reduced systolic blood pressure, TG levels, including total, chylomicron (CM), very LDL (VLDL), HDL-TG, and cholesterol levels, including total, CM, VLDL, and LDL-cholesterol, compared with vehicle treatment. Acetylcholine caused concentration-dependent relaxation of thoracic aorta rings that were pre-contracted with phenylephrine in all rats. Relaxation rates in the HD-vehicle group were significantly lower compared with the ND-vehicle group. Relaxation rates in the HD-combination of pitavastatin and pemafibrate group significantly increased compared with the HD-vehicle group, although neither medication alone ameliorated relaxation rates significantly. Western blotting experiments showed increased phosphorylated endothelial nitric oxide synthase protein expression in aortas from rats in the HD-pemafibrate group and the HD-combination group compared with the HD-vehicle group. However, the expression levels did not respond significantly to pitavastatin alone.

**Conclusions:**

Combination therapy with pitavastatin and pemafibrate improved lipid profiles and endothelial dysfunction in hypertension and insulin resistance model rats. Pemafibrate as an add-on strategy to statins may be useful for preventing atherosclerosis progression.

## Background

Many clinical trials and meta-analyses have revealed that treatment with statins, which are 3-hydroxy-methylglutaryl coenzyme A (HMG-CoA) reductase inhibitors, targets a reduction in low-density lipoprotein cholesterol (LDL-C) and thereby decreases the risk of coronary heart disease (CHD) and all-cause mortality [1]. However, many CHD cases are not prevented and the residual risk factors including high triglyceride (TG) and low high-density lipoprotein cholesterol (HDL-C) levels remain unsettled [2].

Fasting and non-fasting hypertriglyceridemia is a risk factor for CHD [3–5]. Several mechanisms of atherogenesis in hypertriglyceridemia are proposed. Hypertriglyceridemia is involved in the production of proinflammatory cytokines, recruitment of neutrophils, and generation of oxidative stress, resulting in endothelial dysfunction [6–9]. Endothelial dysfunction is an initial process of atherogenesis, and it contributes to the pathogenesis of CHD. Among TG-rich lipoproteins, remnant lipoproteins depress the activity of endothelial nitric oxide synthase (eNOS) in endothelial cells and decrease nitric oxide (NO) released from the endothelium [10, 11].

Endothelial dysfunction assessed by brachial artery flow-mediated dilatation (FMD) has been shown to be impaired in patients with traditional coronary risk factors, including hypertension, dyslipidemia, diabetes mellitus, and smoking, and it has been considered to be a cause of atherosclerosis [5, 6, 12–14]. Statins improve endothelial function as assessed by FMD [15, 16]. In addition, hypertriglyceridemia is independently associated with endothelial dysfunction as assessed by FMD in patients with CHD during statin therapy [17].

Previously, a clinical trial for combination therapy with a statin plus a fibrate to reduce the residual risk was conducted in patients with type 2 diabetes mellitus. The ACCORD study showed that combination therapy with simvastatin plus fenofibrate did not reduce cardiovascular outcomes and mortality compared with simvastatin alone [18]. However, in a preplanned subgroup analysis, there was a trend benefit of fenofibrate in patients with a high TG level (≥ 204 mg/dL) or a low HDL-C level (≤ 34 mg/dL) [2]. Additionally, fibrate treatment during the trial period was associated with a legacy effect of improved survival over a post-trial follow-up [19]. These findings suggest that re-evaluation of TG-lowering therapy as an add-on strategy to statins is needed.

Fibrates activate a transcription factor that belongs to the nuclear receptor superfamily, peroxisome proliferator-activated receptor α (PPARα), and controls lipid metabolism. Recently, pemafibrate (K-877), a novel selective PPARα modulator (SPPARMα), has been developed [20], which is even more potent than fenofibric acid (the active metabolite of fenofibrate) and more specific for human PPARα than either PPARγ or δ [21].

Pemafibrate robustly decreases serum TG levels in fasting and non-fasting or postprandial states and increases serum HDL-C levels [22, 23]. Moreover, pemafibrate was superior to fenofibrate in terms of serum TG-lowering effect and hepatic and renal safety [22]. The ongoing PROMINENT trial is ongoing in patients with type 2 diabetes mellitus, elevated TG, and low levels of HDL-C to determine whether treatment with pemafibrate safely reduces residual cardiovascular risk. Therefore, we hypothesized that combination therapy with a statin plus pemafibrate could notably improve endothelial dysfunction in metabolic syndrome. The aim of this study was to assess the effect of combination therapy with pitavastatin and pemafibrate on lipid profiles and endothelial dysfunction in Dahl salt-sensitive (DS) rats fed a high-salt and high-fat diet, which showed hypertension and insulin resistance [24, 25].

## Methods

### Protocols for animal experiments

**Fig. 1:**
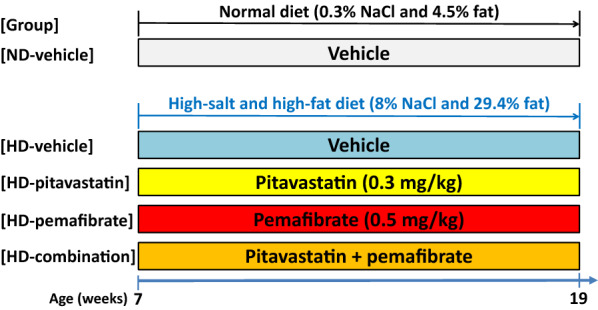
Scheme of experimental protocol. We used 7-week-old male Dahl salt-sensitive rats. Rats were fed a normal diet (ND) (0.3% NaCl and 4.5% fat) or a high-salt and high-fat diet (HD) (8% NaCl and 29.4% fat). The rats were divided into five different groups and treated with vehicle, pitavastatin (0.3 mg/kg), pemafibrate (K-877) (0.5 mg/kg), or a combination of pitavastatin (0.3 mg/kg) and pemafibrate (K-877) (0.5 mg/kg) for 12 weeks. At 19 weeks, blood collection and evaluation of endothelium-dependent relaxations of thoracic aorta were performed

Seven-week-old male Dahl salt-sensitive (DS) rats (n = 44) (Japan SLC, Shizuoka, Japan) were fed a normal diet (ND; 0.3% NaCl and 4.5% fat) (CE-2, CLEA Japan, Inc., Tokyo, Japan) or a high-salt and high-fat diet (HD; 8% NaCl and 29.4% fat) (CLEA Japan), as previously described [26], and they were treated with a vehicle (0.5% carboxy methyl cellulose and 0.5% methyl cellulose), pitavastatin (0.3 mg/kg/day) (Kowa Co., Ltd., Tokyo, Japan), pemafibrate (K-877) (0.5 mg/kg/day) (Kowa Co., Ltd.) or a combination of pitavastatin (0.3 mg/kg/day) and pemafibrate (K-877) (0.5 mg/kg/day) (Fig. 1) by oral gavage for a period of 12 weeks (Fig. 1). Body weight was measured once a week for a period of 12 weeks. Rats were divided into the following five groups: (1) ND-vehicle group (n = 5) fed an ND and treated with vehicle; (2) HD-vehicle group (n = 9) fed an HD and treated with vehicle; (3) HD-pitavastatin group (n = 10) fed an HD and treated with pitavastatin; (4) HD-pemafibrate group (n = 10) fed an HD and treated with pemafibrate; and (5) HD-combination group (n = 10) fed an HD and treated with combination of pitavastatin and pemafibrate. All experimental protocols were approved by and conducted in accordance with the recommendations of the Okayama University Animal Care and Use Committee (permit number OKU-2019349).

### Blood pressure and pulse rate measurement

Systolic blood pressure and pulse rate were measured at 12 and 19 weeks using a tail-cuff plethysmography (MK-2000 Muromachi, Tokyo, Japan or BP-2000, Visitech Systems, Inc., Apex, NC, USA). An average of three measurements was used.

### Blood collection and measurements

At 19 weeks, rats were anesthetized with isoflurane. Non-fasting whole blood was collected from the abdominal aorta into a chilled tube. After centrifugation at 3000×*g* for 10 min at 4 °C, plasma was collected and stored at −80 °C. Plasma total bilirubin was measured using the vanadate oxidase method. Plasma glucose was measured using the hexokinase/glucose-6-phosphate dehydrogenase method. Plasma cholesterol and TG content in lipoprotein fractions including chylomicron (CM), very low-density lipoprotein (VLDL), LDL, and HDL were analyzed using high-performance liquid chromatography by Skylight Biotech (Akita, Japan), as described [27, 28].

### Vascular relaxation studies

Endothelium-dependent relaxation in response to acetylcholine was evaluated. At 19 weeks, rats were anesthetized with isoflurane. The thoracic aorta was rapidly removed, gently cleaned taking care not to damage the endothelium, and it was cut into 3-mm rings. The rings were then cut open. Open aortic rings were placed in a 10-mL organ bath containing Krebs–Henseleit solution (KHS; in mmol/L: 118 NaCl, 4.7 KCl, 2.5 CaCl_2_, 1.2 KH_2_PO_4_, 1.2 MgSO_4_, 25 NaHCO_3_, 11.1 glucose). One end of the open ring was connected to a tissue holder and the other end was connected to a force displacement transducer (AD-611 J, Nihon Kohden, Tokyo, Japan). The bathing solution was gassed with 95% O_2_ and 5% CO_2_ at 37 °C (pH 7.4). The tissue was equilibrated for 60 min under a resting tension of 1 g. During this time, Krebs–Henseleit solution was replaced every 15 min with fresh solution. The tissues were pre-contracted with phenylephrine (0.3 µmol/L). Tissues were then re-washed and pre-contracted with phenylephrine (0.3 µmol/L). After the phenylephrine-induced contraction had reached a plateau, the concentration–response relationships for acetylcholine (1–10,000 nmol/L) were obtained by adding acetylcholine to the bath in a cumulative manner. Finally, papaverine (100 µmol/L) relaxation responses were obtained. The relaxation responses obtained were expressed as a percentage of the maximal relaxation that was evoked by papaverine (100 µmol/L).

### Western blot analysis

Protein samples from the abdominal aortas of 4 or 6 randomly selected rats in each group were prepared using a BEAD crusher (µT-12, Taitec, Koshigaya, Japan). Tissue lysates were extracted in radioimmunoprecipitation (RIPA) buffer with 2 mmol/L phenylmethylsulfonyl fluoride, 1 mmol/L sodium orthovanadate, and 10 mmol/L sodium fluoride (sc-24948, Santa Cruz, Dallas, Texas, USA) and 20 µg of lysates was subjected to SDS-PAGE. Rabbit anti-eNOS antibody (#610297, BD Biosciences, Franklin lakes, New Jersey, USA), rabbit anti-phospho-eNOS (Ser1177) antibody (#612393, BD Biosciences, Franklin lakes, New Jersey, USA), and mouse anti-beta actin antibody (ab6276, Abcam, Cambridge, UK) were used. All primary antibodies were used at a dilution of 1:1000. The second antibody was horseradish peroxidase-conjugated anti-rabbit or anti-mouse IgG antibody (NA934 and NA931, GE Healthcare Bio-Sciences, Buckinghamshire, England). Positive signals were detected using a chemiluminescence system (ECL plus, GE Healthcare Bio-Sciences).

### Quantitative real-time polymerase chain reaction (qPCR) analysis

For reverse transcription (RT)- PCR analysis, RNA was extracted from the abdominal aortas of 4 or 6 randomly selected rats in each group with RNeasy Mini Kit (Qiagen). The total RNA (2 μg) from each tissue sample was used to generate complementary DNA (cDNA) with ReverTra Ace (TOYOBO, Osaka, Japan). The cDNA was subjected to PCR with TaqMan Gene Expression Master Mix (Applied Biosystems, Foster City, CA, USA) and predesigned gene specific primer and probe sets (TaqMan Gene Expresso in Assays; Applied Biosystems). Quantitative real-time PCR was performed using the Applied Biosystems 7300 realtime PCR System (Applied Biosystems). The PCR primers used were the following: NADPH oxidase-4 (NOX4), Rn00585380 and actin beta (ACTB), Rn00667869. ACTB was used as the internal control.

### Statistical analysis

Statistical analysis was performed using SPSS version 24 (IBM, New York, USA). All results are expressed as the mean ± standard deviation (SD). For comparison between different treatment groups, statistical analysis was performed using a one-way analysis of variance (ANOVA) with a Bonferroni post hoc test. Vascular relaxation studies were analyzed using a mixed effect model with a Bonferroni post hoc test. P-values < 0.05 were considered to be significant.

## Results

### Effects of pitavastatin, pemafibrate, or a combination of pitavastatin and pemafibrate on blood pressure and heart rate

After feeding for 12 weeks, systolic blood pressure was significantly higher in the HD-vehicle group compared with the ND-vehicle group (Table 1). After treatment for 12 weeks, systolic blood pressure was significantly lower in the HD-combination group compared with the HD-vehicle group, the HD-pitavastatin group, and the HD-pemafibrate group (Table 1). We observed no difference in heart rate among the five groups at 12 weeks post-feeding of ND or HD and treatment.

**Table 1:** Effects of pitavastatin, pemafibrate or combination of pitavastatin and pemafibrate on BP, HR and blood measurements

Group	ND-vehicle	HD-vehicle	HD-pitavastatin	HD-pemafibrate	**HD-combination**
Food	ND	HD	HD	HD	HD
Treatment	Vehicle	Vehicle	Pitavastatin	Pemafibrate	Combination
Number of rats	5	9	10	10	10
SBP (mmHg)	143 ± 2	194 ± 7*	197 ± 8	193 ± 8	179 ± 10*#§†
HR (beats/min)	376 ± 11	402 ± 37	383 ± 19	400 ± 24	383 ± 31
Blood measurements					
Total bilirubin (mg/dL)	0.01 ± 0.01	0.01 ± 0.01	0.03 ± 0.03	0.01 ± 0.01	0.02 ± 0.01
Glucose (mg/dL)	241 ± 20	236 ± 30	232 ± 22	236 ± 15	228 ± 15

Effects of pitavastatin, pemafibrate, or a combination of pitavastatin and pemafibrate on total and phospho-eNOS and NOX4 expression in the rat aorta

Values are mean ± SD. *P < 0.001 vs. ND-vehicle group. #P < 0.005 vs. HD-vehicle. §P < 0.001 vs. HD-pitavastatin group. †P < 0.01 vs. HD-pemafibrate group. ‡P < 0.005 vs. HD-vehicle

*BP* blood pressure, *ND* normal diet, *HD* high-salt and high-fat diet, *SBP* systolic blood pressure, *HR* heart rate

### Effects of pitavastatin, pemafibrate, or a combination of pitavastatin and pemafibrate on blood measurements

**Fig. 2:**
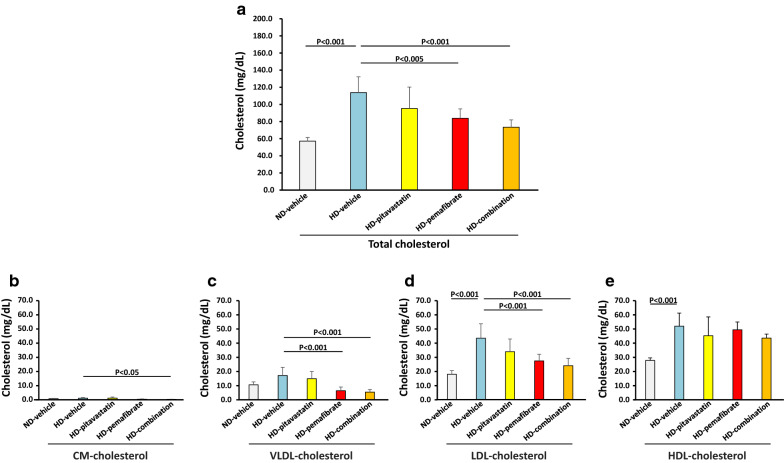
Effects of pitavastatin, pemafibrate, or a combination of pitavastatin and pemafibrate on plasma cholesterol profile. Rats were fed a normal diet (ND) or a high-salt and high-fat diet (HD). The rats were divided into five groups and treated with vehicle, pitavastatin, pemafibrate (K-877), or a combination of pitavastatin and pemafibrate. **a** Total cholesterol, **b** chylomicron (CM)-cholesterol, **c** very low-density lipoprotein (VLDL)-cholesterol, **d** low-density lipoprotein (LDL)-cholesterol, **e** high-density lipoprotein (HDL)-cholesterol. Data are expressed as the mean ± SD. Number of rats in each group: ND-vehicle, n = 5; HD-vehicle, n = 9; HD-pitavastatin, n = 10; HD-pemafibrate, n = 10; and HD-combination, n = 10. Statistical analysis was performed using a one-way ANOVA with a Bonferroni post hoc test

After feeding for 12 weeks, plasma levels of total cholesterol were significantly higher in the HD-vehicle group compared with the ND-vehicle group (Fig. 2a). For lipoprotein fractions, plasma LDL-cholesterol and HDL-cholesterol levels were significantly higher in the HD-vehicle group compared with the ND-vehicle group (Fig. 2c–e).

Treatment with pemafibrate and a combination of pitavastatin and pemafibrate significantly reduced plasma total cholesterol levels compared with vehicle treatment (Fig. 2a). For lipoprotein fractions, plasma CM-cholesterol, VLDL-cholesterol, and LDL-cholesterol levels were significantly lower in the HD-combination group compared with the HD-vehicle group (Fig. 2b–d). Plasma VLDL-cholesterol and LDL-cholesterol levels were also significantly lower in the HD-pemafibrate group compared with the HD-vehicle group (Fig. 2c, d).

**Fig. 3:**
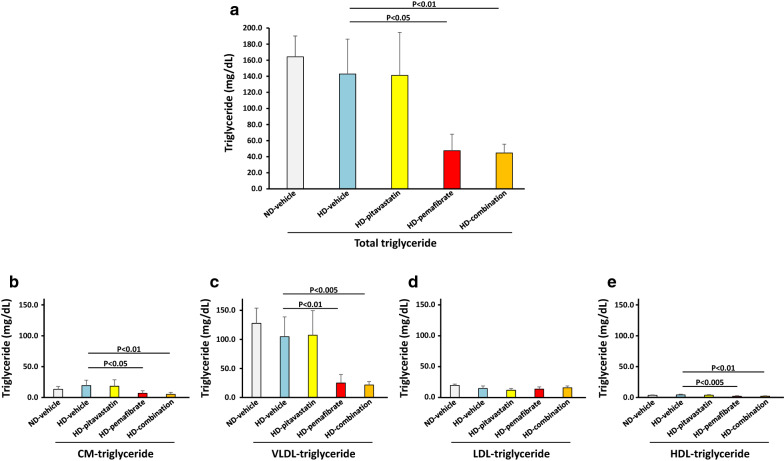
Effects of pitavastatin, pemafibrate, or combination of pitavastatin and pemafibrate on plasma triglyceride profile. Rats were fed a normal diet (ND) or a high-salt and high-fat diet (HD). The rats were divided into five groups and treated with vehicle, pitavastatin, pemafibrate (K-877), or combination of pitavastatin and pemafibrate. **a** Total triglyceride (TG), **b** chylomicron (CM)-TG, **c** very low-density lipoprotein (VLDL)-TG, **d** low-density lipoprotein (LDL)-TG, **e** high-density lipoprotein (HDL)-TG. Data are expressed as the mean ± SD. Number of rats in each group: ND-vehicle, n = 5; HD-vehicle, n = 9; HD-pitavastatin, n = 10; HD-pemafibrate, n = 10; and HD-combination, n = 10. Statistical analysis was performed using a one-way ANOVA with a Bonferroni post hoc test

There were no significant differences in plasma total TG levels between the ND-vehicle group and the HD-vehicle group at 12 weeks post-feeding in accordance with previous reports [24, 25] (Fig. 3a).

Treatment with pemafibrate and a combination of pitavastatin and pemafibrate significantly reduced plasma total TG levels compared with vehicle treatment (Fig. 3a). For lipoprotein fractions, plasma CM-TG, VLDL-TG, and HDL-TG levels were significantly lower in the HD-pemafibrate group and the HD-combination group compared with the HD-vehicle group (Fig. 3b, c, e).

There were no significant differences in plasma total bilirubin and plasma glucose levels among the five groups at 12 weeks post-feeding and treatment.

### Effects of pitavastatin, pemafibrate, or a combination of pitavastatin and pemafibrate on endothelium-dependent vascular relaxation in response to acetylcholine

**Fig. 4:**
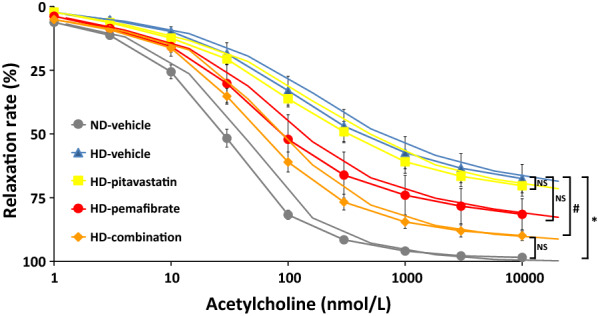
Effects of pitavastatin, pemafibrate or combination of pitavastatin and pemafibrate on endothelium-dependent vascular relaxations in response to acetylcholine. Rats were fed a normal diet (ND) or a high-salt and high-fat diet (HD). The rats were divided into five groups and treated with a vehicle, pitavastatin, pemafibrate (K-877), or combination of pitavastatin and pemafibrate. Data are expressed as the mean ± SD. *P < 0.001, HD-vehicle group versus ND-vehicle group.# P < 0.05, HD-combination group versus HD-vehicle group. Number of rats in each group: ND-vehicle, n = 4; HD-vehicle, n = 5; HD-pitavastatin, n = 5; HD-pemafibrate, n = 5; and HD-combination, n = 5. Statistical analysis was performed using mixed effect model with a Bonferroni post hoc test

Acetylcholine (1 − 10,000 nmol/L) caused concentration-dependent relaxation in thoracic aorta rings that were pre-contracted by phenylephrine (0.3 µmol/L) in all five groups (Fig. 4). Relaxation rates in the HD-vehicle group were significantly lower compared with the ND-vehicle group. Relaxation rates in the HD-combination of pitavastatin and pemafibrate group significantly increased compared with those in the HD-vehicle group, although neither medication alone ameliorated relaxation rates significantly.

**Fig. 5:**
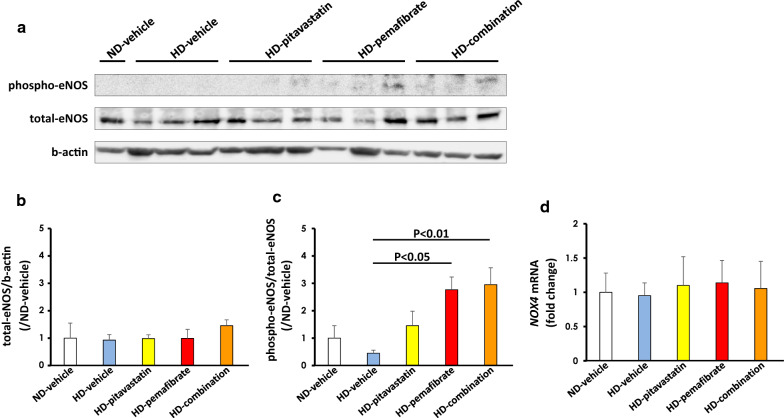
Effects of pitavastatin, pemafibrate, or combination of pitavastatin and pemafibrate on total and phospho-eNOS and NOX4 expression in the rat aorta. **a** Images of Western blot analysis of total and phospho-endothelial nitric oxide synthase (eNOS) in aorta from rats fed a high-salt and high-fat diet (HD). **b** Western blot analysis of total eNOS. **c** Western blot analysis of phospho-eNOS. **d** qPCR analysis of NOX4 mRNA. Data are presented relative to ND-vehicle and expressed as the mean ± SD. Number of rats in each group: ND-vehicle, n = 4; HD-vehicle, n = 6; HD-pitavastatin, n = 6; HD-pemafibrate, n = 6; and HD-combination, n = 6. Statistical analysis was performed using a one-way ANOVA with a Bonferroni post hoc test

Western blotting experiments revealed that there was no significant difference in total eNOS protein expression between aortas from rats of HD-vehicle, HD-pitavastatin, HD-pemafibrate, and HD-combination groups (Fig. 5a, b). Expression levels of phospho-Ser1177 eNOS/total eNOS in the HD-pemafibrate group and the HD-combination group were significantly increased compared to those in HD-vehicle group (HD-vehicle group versus HD-pemafibrate group, P < 0.05; and HD-vehicle group versus HD-combination group, P < 0.01; Fig. 5a, c). However, the expression levels did not respond significantly to pitavastatin alone.

We also investigated the expression levels of NOX4, an enzyme that produces superoxide and plays a pivotal role in generation of oxidative stress in vascular smooth muscle cells [29]. There were no significant differences in expression levels of NOX4 mRNA among the five groups (Fig. 5d).

## Discussion

The major new finding of this work is that combination therapy with pitavastatin and pemafibrate can improve endothelial dysfunction in hypertension and insulin resistance model rats, although neither medication alone ameliorated endothelial function significantly. Additionally, combination therapy significantly reduced systolic blood pressure, TG levels including total, CM, VLDL, and HDL-TG, and cholesterol levels, including total, CM, VLDL, LDL-cholesterol, compared with vehicle treatment and increased expression of phosphorylated eNOS proteins in aortas. These results are considered to be a possible cause of the beneficial effects of combination therapy on endothelial function.

A double-blind, placebo-controlled, phase 2 clinical trial revealed that pemafibrate decreased TG, VLDL-cholesterol, CM-cholesterol, remnant lipoprotein cholesterol, apolipoprotein B, and apolipoprotein C-III levels and increased levels of HDL-cholesterol levels in dyslipidemic patients with high TG and low HDL-cholesterol [30]. This study also showed that pemafibrate decreased TG, including total, CM, VLDL, and HDL-TG, and cholesterol, including total, VLDL, and LDL, in dyslipidemic rats with high TG and high cholesterol levels. A recent meta-analysis also revealed that pemafibrate significantly reduced TG levels [31]. Takei et al. reported that pemafibrate (K-877) is a potential PPARα-modulating drug to treat hyperlipidemia that works well in both the liver and small intestine of LDL receptor knockout (Ldlr^−/−^) mice [32]. Sairyo et al. reported that pemafibrate (K-877) decreased intestinal mRNA expression of ApoB and Npc1l1 [33]. Our study also revealed that pemafibrate improved VLDL-TG and CM-TG, and thus, we presume that pemafibrate had beneficial effects on the liver and small intestine in our model. Because the model showed high HDL-cholesterol levels rather than low HDL-cholesterol levels, pemafibrate did not increase the HDL-cholesterol levels.

It is clinically known that pitavastatin lowers LDL cholesterol and increases HDL cholesterol and that pemafibrate lowers TG and increases HDL cholesterol. However, these effects were not observed in this study except for reductions in TG and VLDL-TG using Pemafibrate. HMG-CoA reductase inhibitors (statins) do not lower plasma cholesterol in rats [34]. In accordance with previous studies, pitavastatin did not reduce cholesterol levels significantly in this study with a rat model. Pemafibrate increased HDL cholesterol levels in mice [35], but our study showed that pemafibrate increased HDL cholesterol levels in DS rats. These results needed to be confirmed in future studies. However, combination therapy with pitavastatin and pemafibrate significantly decreased TG levels, including total, CM, VLDL, and HDL-TG, and cholesterol levels, including total, CM, VLDL, and LDL, in dyslipidemic rats with high TG and high cholesterol. A double-blind, placebo-controlled clinical trial also revealed that pemafibrate add-on therapy in combination with pitavastatin treatment showed a robust reduction of TG in patients with dyslipidemia [36]. These results support the favorable effects of combination therapy on lipid profiles in dyslipidemic conditions.

Combination therapy with pitavastatin and pemafibrate significantly improved systolic blood pressure compared with vehicle treatment in salt-sensitive hypertensive rats fed a high-salt and high-fat diet. Huang et al. reported that endothelium-derived relaxing factor activity, as assayed by acetylcholine-induced relaxation, is absent in eNOS mutant mice and that eNOS mutant mice are hypertensive [37]. Therefore, increased expression of phospho-eNOS on Ser1177 proteins seems to be associated with improvement of blood pressure in this study. Saka et al. reported that treatment with pitavastatin (0.3 mg/kg/d) did not lower blood pressure and levels of TC and TG in DS rats, in accordance with the results of our study [38]. Terata et al. reported that Wistar-Kyoto rats that received L-nitro-arginine methyl ester (L-NAME), a NOS inhibitor, showed a progressive increase in systolic arterial blood pressure and that treatment with a high dose of pitavastatin (1 mg/kg/d) significantly lowered blood pressure. However, levels of TC, TG and HDL cholesterol remained unchanged. Therefore, improvement of lipid profiles in combination therapy might not be involved in the effects of lowering blood pressure in our study [39]. Gilbert et al. also reported that the PPARα agonist fenofibrate lowers blood pressure in salt-sensitive hypertensive patients [40]. The precise mechanism of this phenomenon remains unclear, and further studies are needed to clarify this point.

As for the dose of pemafibrate in animal experiments, Hennuyer et al. reported that pemafibrate at 0.1 mg/kg/day and 1 mg/kg/day reduced plasma TC (−72% and −79% respectively) and TG (−68% and −85% respectively) concentrations compared to the control in human apolipoprotein E2 knock-in (apoE2KI) mice fed a western diet [35]. Dong et al. reported that a high-fat diet reduced the expression of ATP-binding cassette transporter A1 (ABCA1) in the mouse pancreas but that pemafibrate (K-877) treatment (0.3 mg/kg/day) increased ABCA1 expression compared to that in high-fat diet-fed mice [41]. Therefore, we assumed that pemafibrate at 0.5 mg/kg/day could improves lipid metabolism in an animal model fed a high-fat diet.

Increased expression of phospho-eNOS on Ser1177 proteins was observed in aortas from rats in the HD-combination group compared with those from rats from the HD-vehicle group. Because Ser1177 phosphorylation has been shown to increase NO production from eNOS [42], this reaction might have contributed to the improvement of endothelium-dependent relaxation of the aorta in response to acetylcholine using combination therapy. There were no significant differences in expression levels of NOX4 mRNA among the five groups. Other molecules or genes also need to be investigated in future studies.

Regarding the issue of statins and glucose level, Kim et al. reported that use of atorvastatin, rosuvastatin, pitavastatin and simvastatin had a significant association with increase in fasting glucose of non-diabetic individuals [43]. However, Jeong et al. reported that administration of highest-dose pitavastatin did not increase the risk of new-onset diabetes in patients at high risk of developing diabetes [44]. It has been shown that pemafibrate suppresses diet-induced obesity in mice and improves their obesity-related metabolic abnormalities including glucose, insulin and TG levels [45]. We also showed that combination therapy of pitavastatin and pemafibrate did not increase glucose levels. Thus, combination therapy of pitavastatin and pemafibrate can be safely used in patients with metabolic syndrome who are at a high risk of developing diabetes.

### Study limitations

This study has several limitations. First, we only assessed the effects of combination therapy on endothelium-dependent relaxation of the aorta in response to acetylcholine. Other critical homeostatic roles of the endothelium were not assessed in this study. Second, this study was performed using Dahl salt-sensitive rats fed a high-salt and high-fat diet. This model shows hypertension, insulin resistance and endotherial dysfunction, but TG levels are not elevated. Therefore, we could not confirm the effects of an increase in TG-rich lipoproteins or remnant lipoproteins on endothelial dysfunction. Third, the sample size of the ND-vehicle group was smaller than the sample sizes of other groups for a financial reason. Further studies are needed to clarify these points.

## Conclusions

Combination therapy with pitavastatin and pemafibrate improved lipid profiles including TG and cholesterol levels and ameliorated endothelial dysfunction. There was also an increase in the expression level of phospho-eNOS on Ser1177 protein in hypertension and insulin resistance model rats. Pemafibrate as an add-on strategy to statins may be useful for preventing atherosclerosis progression.

## Data Availability

The datasets used and/or analyzed during the current study are available from the corresponding author upon reasonable request.

## Abbreviations

HMG-CoA: 3-hydroxy-methylglutaryl coenzyme A
LDL-C: Low-density lipoprotein cholesterol
CHD: Coronary heart disease
TG: Triglyceride
HDL-C: High-density lipoprotein cholesterol
eNOS: Endothelial nitric oxide synthase
NO: Nitric oxide
RLP-C: Remnant-like particles-cholesterol
FMD: Flow-mediated dilatation
PPARα: Peroxisome proliferator-activated receptor α
SPPARMα: Selective PPARα modulator
DS: Dahl salt-sensitive
ND: Normal diet
HD: High-fat diet
CM: Chylomicron
VLDL: Very low-density lipoprotein
LDL: Low-density lipoprotein
HDL: High-density lipoprotein
KHS: Krebs–Henseleit solution
RIPA: Radioimmunoprecipitation
phospho-eNOS: phosphorylated eNOS
qPCR: Quantitative real-time polymerase chain reaction
Ldlr^−/−^: LDL receptor knockout
