# Correction to: Combination therapy with pemafibrate (K-877) and pitavastatin improves vascular endothelial dysfunction in dahl/salt-sensitive rats fed a high-salt and high-fat diet

**DOI:** 10.1186/s12933-020-01181-7

**Published:** 2020-12-14

**Authors:** Masatoki Yoshida, Kazufumi Nakamura, Toru Miyoshi, Masashi Yoshida, Megumi Kondo, Kaoru Akazawa, Tomonari Kimura, Hiroaki Ohtsuka, Yuko Ohno, Daiji Miura, Hiroshi Ito

**Affiliations:** 1 Department of Cardiovascular Medicine, Okayama University Graduate School of Medicine, Dentistry and Pharmaceutical Sciences, 2-5-1 Shikata-cho, Kita-ku, Okayama, 700-8558 Japan; 2 Department of Medical Technology, Kawasaki College of Allied Health Professions, Okayama, Japan; 3 Department of Basic and Clinical Medicine, Nagano College of Nursing, Nagano, Japan

## Correction to: Cardiovasc Diabetol (2020) 19:149 https://doi.org/10.1186/s12933-020-01132-2

Following publication of the original article [1], the authors identified an error in Fig. 4. The correct Fig. 4 is given.

**Fig. 4:**
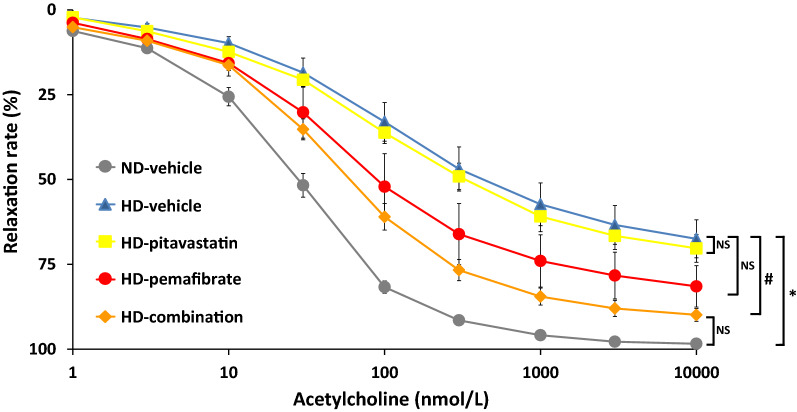
Effects of pitavastatin, pemafibrate or combination of pitavastatin and pemafibrate on endothelium-dependent vascular relaxations in response to acetylcholine. Rats were fed a normal diet (ND) or a high-salt and high-fat diet (HD). The rats were divided into five groups and treated with a vehicle, pitavastatin, pemafibrate (K-877), or combination of pitavastatin and pemafibrate. Data are expressed as the mean ± SD. *P < 0.001, HD-vehicle group versus ND-vehicle group. ^#^P < 0.05, HD-combination group versus HD-vehicle group. Number of rats in each group: ND-vehicle, n = 4; HD-vehicle, n = 5; HD-pitavastatin, n = 5; HD-pemafibrate, n = 5; and HD-combination, n = 5. Statistical analysis was performed using mixed effect model with a Bonferroni post-hoc test

The original article has been corrected.
